# Nutritional Characteristics, Sites of Origin, and Cost of Foods Consumed during School Hours and Their Relationship to Nutritional Status of Schoolchildren in Mexico City

**DOI:** 10.3390/life11050439

**Published:** 2021-05-14

**Authors:** Gloria Martínez-Andrade, Marco González-Unzaga, Guillermina Romero-Quechol, Eugenia Mendoza, Jenny Vilchis-Gil, Ximena Duque

**Affiliations:** 1 Epidemiological and Health Services Research Unit, Mexican Institute of Social Security, Mexico City 06720, Mexico; gloria.martineza@imss.gob.mx (G.M.-A.); marcounzaga@gmail.com (M.G.-U.); guillequechol@gmail.com (G.R.-Q.); 2 Autonomous University of Hidalgo State, Tilcuautla Hidalgo 42160, Mexico; 3 Infectious Diseases Research Unit, Pediatric Hospital, Mexican Institute of Social Security, Mexico City 06720, Mexico; eugenia.mo13@hotmail.com; 4 Epidemiological Research Unit in Endocrinology and Nutrition, Hospital Infantil de México Federico Gomez, Ministry of Health (SSA), Mexico City 06720, Mexico; 5 Medicine Faculty, National Autonomous University of Mexico, Mexico City 06720, Mexico

**Keywords:** schoolchildren, food consumption, school meals, food price, caloric intake, nutritional status

## Abstract

Access, nutritional characteristics, preferences, and cost can affect food intake at school. A cross-sectional study was performed to determine the nutritional characteristics, sites of origin, and cost of foods consumed during school hours. Three hundred and sixty-nine children from five public elementary schools in Mexico City participated. The children gave information about the foods that they consumed five days out of the week during school hours, including the place of acquisition, cost of the food, and portion size. Anthropometric measurements of height and weight of the children were taken. Caloric consumption and percentage of recommended daily energy intake from food during school hours was determined. Children were 10.9 ± 0.9 years old; 55.6% were girls, 26% were overweight, 23% were obese, and 3.3% were of low height for age. The average calorie intake was 515 kilocalories (kcal) (boys, 535 kcal; girls, 476 kcal, *p* = 0.051); calorie intake was higher when school meal intakes included foods from home, school, and outside of school. No significant differences were found in calorie intake by children’s nutritional status. The cost in Mexican pesos per 100 kcal consumed showed differences according to the nutritional status of the children; it was 4.0 Mexican pesos for children with normal weight and 4.2 and 3.8 pesos in children who were overweight or obese, respectively. The information obtained in this study should be used to provide nutritional guidance. The food portion size intake during school hours should be reduced, and the food should come from one or at most two sites, because each extra food represents an increase in the total kilocalorie intake.

## 1. Introduction

Overweightness and obesity have increased in the last few decades, both in developed and developing countries. Researchers are studying the factors that might be related to these increases [1,2]. In Mexico, the latest health and nutrition surveys (ENSANUT) report a high prevalence of overweightness and obesity in schoolchildren (34.4% in 2012 and 35.6% in 2018) [3,4]. Childhood obesity is associated with metabolic, gastrointestinal, respiratory, and orthopedic diseases, and may cause psychological disorders; it represents one of the main priorities for the health sector [5,6]. Schoolchildren gradually adopt dietary habits influenced by family habits, individual preferences, access to food, publicity, and food cost [7]. Children spend most of their time at home and school; the average time per day they are in school is five hours. During school hours, they can eat food brought from home or food they buy before entering school [8,9]. ENSANUT 2006 noted that dietary patterns of schoolchildren included the consumption of sweetened cereals, cakes, sugared beverages, and whole and sweetened milk, all associated with overweightness and obesity [10]. In a study of Mexican children in 2008, it was reported that, during school hours, children consumed an average of 560 kcal (31% of the daily energy recommendation); foods rich in simple carbohydrates and fats predominated, while the consumption of fruits and vegetables was low. Decisions on food consumption were influenced by food access, preferences, cost, and money available [11,12]. According to a study on the data of ENSANUT 2012, for each extra snack in addition to main meals, schoolchildren consumed between 191 to 289 kcals more [13].

In 2010, guidelines were established regarding the sale or distribution of food in stores or cooperatives in public schools [14]. The effect of these guidelines was evaluated in children 9–10 years old; baseline results showed that the energy from foods consumed in school meals was lower than recommended. The energy recommended for a school meal is 276 kcal, which represents 17.5% of the 1579 kcal recommended per day for children aged 6–11 engaged in moderate physical activity. However, it was reported that, between the first and second stage of implementation of the guidelines, energy intake increased 21% (239 to 290 kcal) and surpassed the energy recommendation for the school meal [15]; therefore, continuous follow-up is required and, if necessary, a rethinking of strategy.

Cost is an important determinant in food purchase, and the selection of foods based on cost can affect the quality of a diet [16]. Drewnowski (2010) determined the cost of calories from various groups of foods that form part of the diet of the American population; the results of the study showed that cereals and sugars offer lower-cost energy than fruits and vegetables, and that the portion of foods with high energy content was cheaper. The lower cost of foods with high energy density but low nutrient content was consumed more by groups with lower income and of lower academic levels [17]. However, the results can differ depending on availability, access and cost, level of food processing, climatic conditions, and agricultural production in the different countries.

In studies on the evolution of food prices at the national level, industrialized foods (i.e., processed and ultra-processed) have begun to be cheaper than fresh foods (i.e., unprocessed or minimally processed) [18], which means that processed or ultra-processed foods may be selected preferentially by low-income populations, which is a health risk. 

The object of this study was to describe the nutritional characteristics, sites of origin, and cost of foods consumed during school hours, and the relationship of these with the nutritional status of schoolchildren in Mexico City.

## 2. Materials and Methods

### 2.1. Study Design and Study Population

A cross-sectional study was performed based on a survey of children from five public elementary schools in Mexico City from May to September 2017. The selection of the schools was by convenience; it included boys and girls in the fifth and sixth grades, aged 10–12 years, who had not presented diseases that might modify their diet. Supervision of the school zone and principals of the selected schools authorized the study. Parents and children were informed of the objectives and procedures of the study and gave consent in writing (parents) and assent (children) to participate in the study. The protocol was approved by the Comités de Investigación y de Ética del Instituto Mexicano del Seguro Social, registration no. 2015-785-066.

### 2.2. Measurements

The information obtained consisted of the family’s socioeconomic data, child food consumption during school hours, and anthropometric measurements.

#### 2.2.1. Food Eaten by Children during School Hours

The variables of food were defined as: (1) the consumption of foods during school hours, i.e. foods that the child consumed inside of school and on the way to and from school and home (this information was obtained over five days); (2) portion size (amount in g or mL of each food consumed); (3) food origin (place where the food was prepared or bought to be consumed during school hours); (4) energy content of the food (the total amount of kcal from food and the percentage of calories that come from carbohydrates, proteins, and fats that compose the food); and (5) cost of food (amount in national coin assigned to a food by unit of weight, volume portion, or package). One nurse and three nutritionists used standardized measures to evaluate diet.

Information about the foods consumed by the children was obtained through a food frequency questionnaire adapted from the questionnaire for schoolchildren used in the health and nutrition surveys in Mexico [4]. The format of the adapted questionnaire contained four columns: (1) the names and pictures of 118 foods habitually consumed by schoolchildren in Mexico City [19]; (2) the names of the days of the week (Monday to Friday) so that the children could mark the day they consumed each food; (3) an image of the home servings of each food and a space to fill in the number of measures or portions consumed of that food; and (4) food origin sites (home, school, and outside of school). The nutritionists explained to the children how the information would be recorded, using examples of home measurements (plates, spoons, and cups) and food packages as a reference to estimate the amount of food servings consumed.

The children gave information about the foods they consumed over the five weekdays going from home to school, school to home, and inside of school, and about the origin of the foods. The questionnaire was answered on two occasions by the children. The first time, they were asked about consumption over two days; the second time, they were asked about consumption over three days. The information about the five days was recorded in a single file. The interviews with the children were held after the recess period. Nutritionists reviewed the questionnaires with each child after they responded to verify that all data were correctly recorded. Data related to the description, cost, and portion of foods available at home and those acquired inside and outside of school were obtained by research staff through the purchase, weight, and review of the packages of the foods the children said they had consumed. For each food, its characteristics (variety, brand, and type of preparation), the number of portions consumed, the name of the servings (piece, cup, plate, spoonful, package, etc.), amount in g or mL, and the cost (in Mexican pesos) corresponding to each portion were recorded; some of these characteristics were different depending on the origin of the food. The cost per g of each food was obtained by dividing the cost of the portion by the number of g corresponding to the portion. Energy density was the quotient of division of the calories offered by the portion by the quantity of g of the portion.

The information was uploaded and analyzed in The Food Processor SQL version 11.3 software (ESHA Research, Salem, UT, USA). Data were entered by day and place of acquisition; the average in grams of the foods consumed was calculated. Caloric content, macronutrients, and fiber consumed by day and site of acquisition were obtained. Caloric content and the percentage of calories from macronutrients were evaluated in relation with the recommendations proposed by the Secretary of Public Education for Mexican schoolchildren: 1579 kcal/day and 276 kcal from school meals, which represents 17.5% of the daily recommendation. The recommended distribution of the total energy value from macronutrients is 55–60% from carbohydrates, 25–30% from fats, and 10–15% from proteins [14].

#### 2.2.2. Anthropometric Measurements

Anthropometric measurements of height and weight of the children were made according to WHO guidelines. The children wore light clothing without adornments on their head and without shoes; they were asked to stand at the center of the scale or stadiometer with their arms at their sides. Weight was measured with the Seca 878 digital scale (Seca Corp, Hamburg, Germany) with a precision of 0.1 kg, and height with a portable Seca 213 stadiometer (Seca Corp, Hamburg, Germany) with a precision of 0.1 cm. 

The nutritional status was defined using the body mass index (BMI) in z-score and the height for age in z-score using the reference population by age and sex [20,21] and the program Anthro Plus [22]. Nutritional status was classified according to the BMI: low weight < −2.0 SD, normal −2.0 to 1.0 SD, overweight > 1.0 to 2.0 SD, and obese > 2.0 SD, and height for age as low height < −2.0 SD and normal ≥ −2.0 SD [20,23]. 

At the end of the study, the nutritionists provided the participants (children, parents, and teachers) with information about their diagnosis by anthropometry and diet, as well as recommendations for the preparation of healthy school meals.

#### 2.2.3. Sociodemographic Data

Parents responded to a questionnaire about their family’s sociodemographic characteristics. Information on the age and level of education of the mothers and fathers was obtained. We classified this information as less than high school and high school or more. In addition, information was obtained on paid work and marital status (married/common law and single).

### 2.3. Statistical Analysis

The sociodemographic characteristics of the children and their families, food consumption during school hours, and nutritional status based on BMI and height for age indicators of the children are described.

Using medians and interquartile ranges (IQRs), the calories per day obtained from the foods consumed during school hours, the cost per 100 kcal, and the percentage of energy from proteins, carbohydrates, and fats by BMI categories were reported. Statistical comparisons were performed using the Kruskal–Wallis test, Dunn’s test with a Bonferroni correction post hoc due to multiple comparisons, and the Wilcoxon rank sum test.

The percentage of the contribution of food most frequently consumed to energy consumption during the school hours is reported by site of food origin: home, school, or outside of school. The average percentage of the contribution of energy consumption during school hours was calculated from macronutrients using the average weight of the food portion.

From all of the foods consumed during school hours, 57 groups were identified as having similar nutritional characteristics, and the 10 food groups with the highest percentages of consumption by site of origin were described. A calculation was made of the median and IQR of weight or volume, calories, and cost per portion, as well as median cost per g of food, caloric density, and grams of macronutrients and fiber by portion of the food groups.

Values of *p* < 0.05 were considered statistically significant for all of the analyses. The analysis was performed using Stata v14.0 (Stata Corp., College Station, TX, USA).

## 3. Results

A total of 493 fifth and sixth grade schoolchildren were invited to participate in the study, and 371 (75.3%) agreed to participate; the data of two children were eliminated because the calorie intake was outside of the acceptable range (kcal > 3 standard deviations from the general average). The results are presented for 369 children (Table 1), who were an average of 10.9 ± 0.9 years old; 55.6% were girls, 26.0% were overweight, 23.0% were obese, 1.1% had a low weight, and 3.3% had low height for age. 

Mothers of the children were 37.6 ± 6.4 years old, 76.6% were married or common law, 54.0% had a below high school education, and 61.3% had a paying job. Fathers were 41.3 ± 7.5 years old, 54.5% had a below high school education, and 98.6% had a paying job. The median weekly food expense for food consumed in the home was 800 Mexican pesos and 200 pesos for food consumed outside of the home (1 US dollar ≈ 20 Mexican pesos); on average, five people lived in the home. In the comparison by BMI categories, differences were identified in age, sex, and height for the age z-score. The overweight children were older than the schoolchildren with a normal BMI and older than the obese children (*p* = 0.044). Among the schoolchildren with a normal BMI, the percentage of girls was higher (62.5%), and, among the schoolchildren with obesity, 43.5% were girls and 56.5% were boys (*p* = 0.012). The height for age (z-score) was higher in schoolchildren with obesity, followed by children with an overweight BMI and children with a normal BMI (*p* < 0.001).

### 3.1. Energy Contribution during School Hours to Recommended Energy Per Day 

The median calorie intake during school hours was 515 kcal (32.6% of the daily energy intake recommended to schoolchildren); intake was greater in boys (535 kcal) than girls (476 kcal) (*p* = 0.051). The macronutrient distribution was in accordance with the recommendation, and it was not different by nutritional status (Table 2).

The caloric consumption during school hours for children with a normal BMI was 524 kcal, in overweight children it was 467 kcal, and in obese children it was 489 kcal; the difference between caloric intake by children’s nutritional status was not statistically significant.

The cost per 100 kcal consumed by schoolchildren with a normal, overweight, or obese BMI was 4.0, 4.2, and 3.8 Mexican pesos, respectively. The cost/100 kcal was different by nutritional status; these differences were significant between overweight children versus children with a normal BMI and between overweight children versus those with obesity (*p* < 0.05).

There were no significant differences between the calories consumed from each food origin by nutritional status; 52.9% of schoolchildren acquired food from three sites (home, school, and outside of school), 11.4% of children acquired food from school and outside of school, 26.3% from home and school or from home and outside of school, and only 9.5% of children consumed food from only one site (home or school). These percentages were similar by nutritional status (chi^2^ = 6.42, *p* = 0.378). However, caloric consumption was higher when the food originated from three sites, home, school, and outside of school (597 kcal, 37.8% of the daily energy recommendation), rather than when the food was from one site, either home or school (329 kcal) or from two sites, school and outside of school (465 kcal), or home and school or home and outside of school (451 kcal) (*p* < 0.001). Slightly more than half (52.9%) of the children consumed foods from home, school, and outside of school, which offered a median of 597 kcal (IQR: 428, 781), while 9.5% consumed foods that came only from home or school, with a median offering of 329 kcal (IQR: 223, 476) (Table 3).

### 3.2. Food Groups or Preparations More Commonly Consumed by Schoolchildren and Their Nutritional Characteristics

The foods reported by the children were grouped according to similar nutritional characteristics, yielding 57 groups. The most frequently reported 10 groups of foods by origin are presented in Table 4. The number of reports of foods or preparations consumed on five school days was 6161, since the consumption of each food or preparation could be reported once or more during the days evaluated: 33.7% of the reports corresponded to foods brought from home, 49.1% to foods bought at school, and 17.2% to foods bought outside of school.

The food groups and preparations that more than 30% of the children consumed during school hours by food origin were (1) brought from home: fruits, 61.9%; sandwiches, 33.5%; and tortas (salty bread that is filled with various foods), 30.2%. (2) Bought at school: fried foods (mainly popcorn), 51%; fruits, 49.0%; tacos (rolled corn tortilla with some food inside), 45.6%; sorbet, 37.0%; sweets/candy, 33.6%; and vegetables, 31.9%. (3) Bought outside of school: fried foods (wheat flour or potato preparations), 33.5%; and fruits, 31.9%.

**Figure 1:**
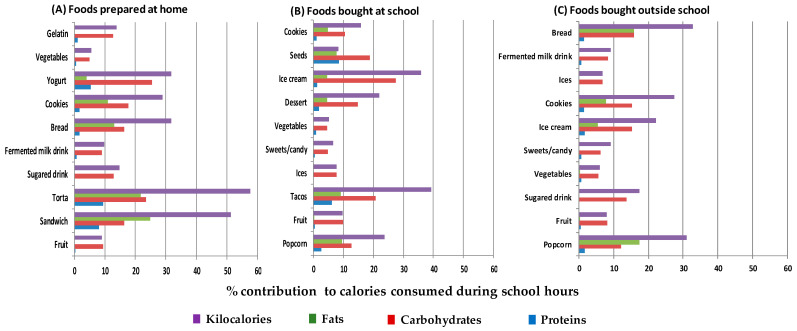
Percentage of total calories and the macronutrient contribution of the food groups and preparations most frequently consumed to caloric intake during school hours. (**A**) Foods prepared at home; (**B**) foods bought in school; and (**C**) foods bought outside school. Torta: salty bread that is filled with various foods; tacos: rolled corn tortilla with some food inside.

Figure 1 shows the 10 food groups or preparations with the highest consumption during school hours by origin, with their respective contribution to the total calorie consumption in school hours and with the percentages of calories obtained from proteins, carbohydrates, and fats from each food or preparation. The preparations with a higher percentage of calorie contribution to the energy consumed during school hours were: tortas and sandwiches from home (more than 50%), ice cream and tacos acquired at school (35.9% and 39.3%, respectively), and fried foods and bread bought outside of school (31.0% and 32.8%).

The foods with the highest percentages of energy from carbohydrates were yogurt brought from home (25.4%), ice cream bought at school (27.5%), and bread bought outside of school (15.9%). The foods with the highest percentages of energy from fat were sandwiches brought from home (24.9%), chips or popcorn from school (9.5%), and fried foods or chips from outside of school (17.4%). The food groups with the highest percentages of contribution to energy consumption from proteins were sandwiches and tortas from home (8.0% and 9.4%, respectively) and tacos and seeds bought at school (6.1% and 8.6%, respectively). The percentages of energy from proteins were low in foods bought outside of school (0.0% to 1.7%), with 1.7% to ice cream.

The tortas and sandwiches brought from home and the tacos and seeds bought at school included ingredients with a high contribution of carbohydrates, fats, and proteins.

### 3.3. Nutritional Contribution, Serving Sizes, and Cost of Food and Preparations More Frequently Eaten by Children during School Hours

The descriptions of the nutritional characteristics (kcal, caloric density, g of proteins, carbohydrates, fats, and fiber) of the portion and cost of the 10 food groups eaten most frequently during school hours by origin are presented in Table 5.

Among the foods from home, sugary beverages represented the portion with the greatest weight, median = 300 g (IQR: 250, 500), and the lowest cost per g, median = 0.01 pesos/g. Cookies represented the smallest portion, median = 37 g (IQR: 24, 60), with a greater caloric density, median = 4.6 kcal/g. Tortas had the highest offering of kcal per portion, median = 328 kcal (IQR: 307, 395). Vegetables had the lowest calories, median = 22 kcal (IQR: 11, 53), the lowest cost per portion, median = 1.8 pesos (IQR: 0.9, 1.8), and the highest content of fiber, median = 2.1 g. Sandwiches cost the most, median = 7.3 pesos (IQR: 5.4, 8.9).

Regarding foods acquired at school, the portion that weighed the most was vegetables, median = 154 g (IQR: 105, 155), and the group that weighed the least was sweets and candies, median = 8 g (IQR: 7, 14).

Vegetables had the least calories, median = 21 kcal (IQR: 14, 59), a lower cost per g, median = 0.03 pesos/g, and lower energy density, median = 0.4 kcal/g. Sweets and candies had less cost per portion, median = 3.0 pesos (IQR: 3.0, 5.0); however, the cost per g, median = 0.4 pesos/g, and the energy density, median = 3.9 kcal/g, were the highest. Tacos had a higher number of calories per portion, median = 186 kcal (IQR: 126, 297). Fruits, like vegetables, had a lower cost per g, median = 0.03 pesos/g. Seeds had the highest amount of fiber, median = 3.1 g in a portion of 41 g.

As for the groups of foods acquired outside of school, the portion with the greatest weight was sugared beverages, median = 300 g (IQR: 250, 500), and the portion with the lowest weight was sweets and candy, median = 12 g (IQR: 6, 20). Beverages with sugar had the lowest cost per g, median = 0.01 pesos/g. Vegetables offered the least kilocalories per portion, median = 27 kcal (IQR: 11, 53), had a lower cost per portion, median = 1.8 pesos (IQR: 1.3, 1.8), and per g, median = 0.01 pesos/g, the lowest energy density, median = 0.4 kcal/g, and the highest amount of fiber, mean = 3.4 g. Bread represented the group with the greatest calorie offering, median = 240 kcal (IQR: 224, 260). Sorbet was the most expensive group per portion, mean = 10.0 pesos. Fried foods had the highest energy density, median = 5.5 kcal/g. 

## 4. Discussion

The purpose of this study was to identify and describe the foods consumed by schoolchildren during school hours. Various factors influence the selection of foods that children consume at school and traveling to and from home and school; among these are the organoleptic characteristics of the foods, the size of the portions, price, and the site of purchase or preparation, along with dietary customs or habits [24]. Other factors are the number of hours that the children stay in school, the distance between homes and schools, and the time and types of transportation. The results of this study allow a current diagnosis of food and preparation intake by children during school hours.

Both at the national and international levels, there is a high prevalence of overweightness and obesity in the school population, as well as in adults. Governments have proposed actions focused on the sectors of the population affected to combat this public health problem. However, there are social, economic, cultural, geographical, and structural factors that are determinants in the increase in overweightness and obesity in children, and they involve resistance to the changes that are needed to acquire healthy dietary habits [25]. Therefore, it is necessary to know and understand eating habits and access to food to plan and implement strategies to promote healthy eating.

Studies in Mexican schoolchildren have reported that, during school hours, children consume around 30% of the 1579 kcal recommended daily for their age and sex [12,26,27]; this percentage represents approximately twice the recommendation proposed of 17.5% of the energy value per day for school meals [14,28]. In an educational intervention to modify eating habits aimed at parents and their children in schools in Mexico City, no change was achieved in the caloric content or in the quality of school meals brought from home. However, throughout the one-year follow-up, 50% of the children had school meals with an energy value higher than 340 kcal, representing an excess of energy of 150 to 200 kcal. In that study, only the kcal from the food brought from home were estimated, without taking into account that children can also buy food inside and outside of school [28]. Another study shows that each additional snack or eating occasion is linked to an increase in total daily energy of approximately 191–289 kcal in schoolchildren [13], so there is concern about the excessive amount of energy that school meals can contribute to the total daily diet of children. This suggests that it is imperative to change eating behavior from an early age regarding the frequency, quantity, and quality of food consumed during school hours. In our study, the median calorie consumption during school hours was 515 kcal (32.6% of the daily energy intake recommended), and it was characterized by including foods that came from home, school, and outside of school. If the excessive calorie consumption during school hours occurs constantly during all weekdays and there is no redistribution of the calorie offering from subsequent meals that children eat each day, the children’s BMI will increase [29,30].

### 4.1. Percentage of Kilocalories Provided by Food during School Hours Is Higher if it Includes Food from Two or Three Origins

In our study, the school meals that included only foods brought from home or only foods bought inside of school had the lowest calorie count. Our results are similar to those reported in a study that evaluated the effect of the third stage of implementation of the guidelines for the sale of foods in schools in Mexico, since the lowest intake was from foods bought in school (290 kcal), and the highest caloric consumption was from foods that included both origins, those brought from home and those bought inside of school (474 kcal) [15]. However, as mentioned before, in our study, 52% of the children included foods from three sites: home, school, and outside of school, which provided 38% of the daily energy intake recommendation for Mexican schoolchildren. The above offers evidence to recommend that school meals include foods from a single site, whether brought from home or acquired in school. In either case, the meals should include a portion of fruits or vegetables, a prepared food (e.g., a sandwich, torta, or quesadilla, which combines energy sources, such as cereals, bread, or tortilla; a protein source, such as eggs, meat, or cheese; and vegetables), and drinking water [31]. 

### 4.2. Consumption of Fruits and Vegetables during School Hours

One outstanding characteristic in our study of foods included in school meals was that most had fruits, either from home or bought in school or when leaving school, which indicates their availability, that they are well-accepted by the children, that their consumption as part of the school meal is a habit, or that various orientation interventions have impacted this aspect [32,33,34,35]. In contrast, vegetables were seldom included, and were consumed more frequently when bought in school rather than when brought from home; vegetables have high fiber and low calories, so it is convenient to include them every day in the school meal. The fact that vegetables are not brought from home and are bought in school again suggests an impact of the strategies regarding the availability of this type of food in schools. It is also possible that they are not brought from home because it is difficult to keep them fresh inside a lunchbox or because parents do not have enough time to buy and prepare them. This aspect should be corroborated in other studies, since it might be possible to provide thermic lunchboxes, have school refrigerators to store the lunchboxes, or promote the sale of vegetables inside of schools, taking advantage of the Mexican population´s custom of eating jicama, cucumber, and carrots with lemon and chili for snacks.

The inclusion of fruit in our study was similar to that reported in a group of children from Mexico City, where 75% included fruit and vegetables in the school meals [36]; even in our study, this percentage was higher, since we categorized fruits and vegetables separately. However, the percentage of school meals that included fruit and vegetables brought from home or acquired in school was lower than reported for children in northern California, USA, which, as part of a dietary program in schools, offered fruit in 90% and vegetables in 54% of schools meals [37].

Fruits and vegetables offer health benefits, provide vitamins, minerals, and fiber, and in general are eaten in their natural form, without processing or additional sugars or fats; their consumption is recommended, since they can replace the consumption of products with a high calorie content and low nutrient density that have been associated with an increased risk of obesity in children and development of cardiovascular diseases [38,39].

Different factors of the physical, social, and economic environments are determinants for the consumption of fruit and vegetables by children; availability and accessibility are particularly important. Consumption by the family and schoolmates can motivate the acceptance of these foods [40]. Programs in the schools included in this study have promoted the sale of ready-to-eat fruits and vegetables.

### 4.3. Macronutrient Content of Food and Beverages Consumed during School Hours

Meals brought from home or purchased in school included preparations that combined two or more foods that were sources of energy from carbohydrates, such as bread, tortillas, rice, and two or more food sources of protein and fats, such as sausage, eggs, and meats. One food source of each macronutrient should be included to reduce the high calorie intake. Additionally, some school meals are accompanied by products such as chips, desserts, or sweets. This point should also be taken into consideration, and the availability or access to healthier foods for children, parents, principals, teachers, and food vendors inside and outside of the school should be promoted and regulated [41].

The preparations that children most frequently brought from home were sandwiches and tortas. They have important amounts of proteins because one of their ingredients is a food of animal origin, and they also usually contain some vegetables. In school, children mainly bought vegetables, popcorn, tacos, and seeds; vegetables and popcorn are sources of fiber, and tacos offer adequate amounts of carbohydrates, proteins, and fats. Seeds are a source of protein and fiber, and schoolchildren consumed them in sufficient amounts. Seeds such as sunflower seeds, pistachios, peanuts, and nuts offer fiber; however, “Japanese-style peanuts,” which were consumed the most, have a covering based in flours and sugar that increases the energy content through carbohydrates. The foods acquired most outside of school are fried foods (based on wheat or corn flour, potatoes, and oils for preparation) and sugared beverages, with high calorie content and little or no micronutrient content. Some studies have reported that overweightness and obesity in children coincide with an increased consumption of sweet or salty snacks and a reduction in the nutrients consumed in the three main meals. These snacks, chips, and foods with a high sugar content are available at various sites in the children’s surroundings, and have been associated with a reduction in feeling satiated [42].

Some of the food groups brought from home in this study are the same as those described in a study of third to sixth grade students in an elementary school in Tijuana (a border city between Mexico and the United States) [43]; however, the percentages of consumption are different. Our study reported a greater consumption of fruits and slightly lower consumption of tortas and sandwiches, sugared beverages, yogurt, cookies, bread, and vegetables. In both studies, the foods most reported being acquired in school were chips, which have a high content of carbohydrates and fats. The foods most often bought outside of school in our study were chips and fruits, while, in Tijuana, they were ice cream, sweets, chocolates, cookies, and breads. These differences in consumption may be due to the availability of foods, climactic conditions, and modifications in the sale of foods inside and outside of school. The children in our study reported a higher consumption of fruits and vegetables compared with the consumption identified by ENSANUT 2016 in schoolchildren, which reported that 45.7% of the children ate fruits and 22.6% vegetables; chip consumption was lower in our study than the national level, which reported that 61.9% of schoolchildren consumed them [44].

Some food groups, such as chips (salty snacks), yogurt, cookies, and sorbets or ice cream, that were commonly consumed by schoolchildren stood out for their high energy content from carbohydrates and/or fats. Consumption of these highly processed foods may correspond to children’s customs and habits; however, the size of the portions of these foods should be lower. They should be prepared from natural fruits, and the amount of added sugar should be reduced. There is evidence that ultra-processed foods are nutritionally inferior to unprocessed or minimally processed foods, since they are high in carbohydrates, simple sugars, total fats, saturated and trans fats, colorants, flavorings, additives, and energy density, and are low in vitamins, minerals, and fiber content [45]. It is necessary to promote the consumption of foods that are natural or minimally processed and to propose interventions at the government level to improve sufficient access to these foods (e.g., promote natural beverages with little or no sugar added). 

The consumption of foods high in fats and simple carbohydrates in our study was higher than that reported in ENSANUT 2012, where the calories from products high in saturated fat and/or added sugar was 21% of the energy intake for one day in schoolchildren [46]. However, in national health and nutrition surveys, reporting is done by the mother or tutor, and it is possible that many of the foods that the children buy in or outside of school are not included. Some studies only include foods eaten at school recess and exclude foods consumed going from home to school and vice versa. Mexico City is a complex city due its size and traffic, and many hours are spent on transportation. The children leave early in the morning to go to school, with some of them not having breakfast at home (in this study, 20% of children). They have their lunch at home at 3:00 pm, and they frequently buy foods when they leave school.

### 4.4. Nutritional Characteristics, Portions, and Cost of Foods 

In addition to the nutritional content of foods, portion size, tastes, and cost influence their purchase and consumption. Beverages and dairy products with sugar represent the largest portions, while the cost per g is low. The foods with the highest costs per portion were chips (salty snacks), ice cream, and sorbets, acquired outside of the school. The most expensive products per g were sweets and candies.

In this study, foods available in school have a standard price of 5 Mexican pesos (0.25 dollars) for prepared foods, such as tacos, tortas, and desserts, and for foods such as fruits and vegetables. Items acquired in school, which include higher priced foods, are of animal origin, and the amount used for the food corresponded to a smaller proportion in relation to the total preparation. Foods prepared at home, such as sandwiches, had a higher cost; however, in these, the family could control the quantity and quality of ingredients and the hygiene in preparation, so the recommendation to bring foods and preparations from home continues to be in effect. Furthermore, foods prepared at home had a more balanced content of macronutrients and fiber, and had little processing. The costs of the products sold outside of school were high, which could be determined by the chain of intermediaries and the profit expected. Children could buy foods and consume them at different times during school hours, and the decision to buy might be influenced by food preference, attention-getting packaging, publicity, and the relation of price to product quantity [47]. In the school curriculum, it is necessary to include nutrition and health assignments to study subjects, such as the adequate number of meals a day, the health benefits of a correct diet, and the risks of consuming ultra-processed foods with high kcal content and low nutrient content.

### 4.5. Relationship of Caloric Intake and Cost of Food during School hours with the Nutritional Status of Schoolchildren

In the analysis of the calorie content of foods consumed during school hours, no statistically significant differences were found according to nutritional status; however, it was noted that normal weight children consumed more calories, while children with overweightness or obesity consumed fewer calories. One possible explanation could be underreporting in children who were overweight or obese; it has been found that underreporting of energy intake is more prevalent among children and adolescents with a higher BMI [48,49]. National health surveys, such as NHANES and, in Mexico, ENSANUT, have reported that obese children ingested fewer calories, findings that have been considered by authors as possible underreporting [50,51]. Other possible explanations could be a replacement or redistribution of the amount of calories consumed at home by those consumed during school hours, but we cannot conclude this, since the study did not evaluate the total daily diet of the children. Another aspect that influences nutritional status is the energy spent through physical activity performed, which was not analyzed in this study.

In addition, overweightness and obesity in childhood could be the result of multiple factors, such as dietary habits, consumption of high calorie density foods, physical activity, the adoption of a sedentary lifestyle, changes in socioeconomic conditions, and urbanization [27,52].

In the analysis of the relation between cost and caloric content, it was noted that the cost per 100 kcal of foods consumed was lower in obese children and was different from the cost per 100 kcal of foods consumed by overweight children. The price of foods is a determinant in food consumption, and some studies have reported that industrialized foods may have lower prices than fresh foods, which means that the former may be selected preferentially by the low-income population [53,54].

This study had some limitations. The study proposed to identify food consumption during school hours, and the information came directly from the children. Children had difficulty remembering the foods consumed over five days of school attendance, so the evaluations were done in two interviews, which related consumption over two and three days; the interviews were done after school recess, and foods the children bought on previous days were recorded, leaving school. They were also asked what they would buy when leaving school. To evaluate the total energy consumption per day and know if the child consumed a higher percentage of calories than recommended both during school hours and at home, or if a redistribution of calorie content was done in the various meals, it would be necessary to evaluate dietary consumption over the entire day; this information would be useful, but would include information that the children could not estimate with precision.

## 5. Conclusions

The results of this study allowed us to know, describe, and understand the food consumed during school hours by schoolchildren in Mexico City, including the nutritional characteristics, the foods most frequently consumed, and the amount and cost of the foods, which is useful for the planning and evaluation of strategies aimed at improving dietary habits in the school population. It is important because of the global epidemic of childhood overweightness and obesity; in this study, a high percentage of schoolchildren were overweight (26%) or obese (23.0%).

The school meals that included foods brought or bought only from one site (home or school) had lower calorie content than meals that included foods from two or three sites (home, school, outside of school, and their combinations). However, calorie intake during the school hours had no relation with nutritional status; in addition, the cost of kilocalories consumed by children with obesity was lower. In the participating schools, healthy foods, such as fruit, vegetables, and preparations that included food sources of protein, were available, but also available were foods whose consumption is recommended in low amounts due to their relationship with overweightness and obesity, such as chips (salty snacks), desserts, and sweets with high sugar and fat content, and in portions that should be smaller.

The results of this study could be used by public health policy makers, including authorities from the Secretary of Public Education and the city government, since, in Mexico, there is great interest in promoting a healthier diet to reduce the high prevalence of overweightness and obesity in the Mexican population. The information obtained in this study should be used to provide nutritional guidance. The recommendation will be that food portions should be reduced and/or that the food consumption during school hours should come from one or at most two sites (home and school or home and outside of the school or school and outside of the school), but not from three places, because each extra food represents an increase in the total kilocalorie intake. In addition, as the school stores or cooperatives in public schools must comply with guidelines regarding the sale or distribution of food in stores, the food sold to the school community around schools should also be regulated and monitored under the same guidelines.

## Figures and Tables

**Table 1:** Sociodemographic characteristics of the schoolchildren and their families by nutritional status.

	All Children		Nutritional Status Based on Body Mass Index *						
Characteristics	*n* = 369	100%	Normal ^1^ *n* = 184 (50%)		Overweight ^2^ *n* = 96 (26%)		Obesity ^3^ *n* = 85 (23%)		*p*- Value ^†^
Age (years) (mean ± SD)	10.9 ± 0.9		10.8 ± 0.9		11.0 ± 0.8		10.8 ± 1.0		0.044
Sex (Girls)	205	55.6	115	62.5	51	53.1	37	43.5	0.012
Elementary school grade									
Fifth	198	54.0	102	55.4	48	50.0	47	55.3	0.661
Sixth	171	46.0	82	44.6	48	50.0	38	44.7	
Anthropometry									
Body mass index, z-score (mean ± SD)	0.87 ± 1.3		–0.1 ± 0.7		1.4 ± 0.3		2.5 ± 0.4		<0.001
Height for age, z-score (mean ± SD)	–0.1 ± 1.0		–0.4 ± 1.0		0.0 ± 0.9		0.5 ± 0.8		<0.001
Low height to age ^4^	12	3.3	8	4.4	3	3.1	1	1.2	0.397
Characteristics of the mother (*n* = 253)									
Age (years) (mean ± SD) (*n* = 237)	37.6 ± 6.4		37.4 ± 6.3		38.4 ± 6.4		37.1 ± 7		0.681
School level (*n* = 252)									
Less than high school	136	54.0	72	55.4	32	51.6	30	52.6	0.869
High school or more	116	46.0	58	44.6	30	48.4	27	47.4	
Working (*n* = 253)	155	61.3	82	63.1	34	54.8	36	63.2	0.512
Marital status (*n* = 252)									
Married/common law	193	76.6	102	79.1	48	77.4	41	71.9	0.564
Single	57	23.4	27	20.9	14	22.6	16	28.1	
Characteristics of the father (*n* = 213)									
Age (years) (mean ± SD) (*n* = 213)	41.3 ± 7.5		41.2 ± 7.4		41.9 ± 7.8		41.1 ± 7.7		0.776
School level (*n* = 209)									
Less than high school	114	54.5	59	56.2	26	46.4	27	58.7	0.386
High school or more	95	45.5	46	43.8	30	53.6	19	41.3	
Working (*n* = 211)	208	98.6	107	99.1	54	98.2	45	98.6	0.806
Characteristics of the family (*n* = 247)									
People/home, median (IQR) (*n* = 247)	5 (4, 6)		5(4, 6)		4 (4, 5)		5 (4, 6)		0.176
Weekly family food expenses in Mexican pesos, median (IQR)									
Home (*n* = 211)	800 (600, 1050)		800 (500, 1000)		900 (600, 1100)		800 (600, 1200)		0.642
Outside the home (*n* = 181)	200 (100, 400)		200 (65, 350)		300 (100, 400)		200 (100, 300)		0.242

* Diagnostic based on the z-score of the body mass index; the low weight group with four children is not presented. ^1^ Normal: –2.0 to 1.0 SD; ^2^ overweight: > 1.0 to 2.0 SD; ^3^ obesity: > 2.0 SD. ^4^ Diagnostic based on the z-score for height for age: < –2.0 SD. ^†^ Kruskal–Wallis test and Pearson’s chi-squared test. IQR: interquartile range.

**Table 2:** Calorie consumption and cost/100 kcal by nutritional status and sex in schoolchildren in Mexico City.

Contribution to Daily Energy Intake Recommended and Macronutrients Distribution *	All Children *n* = 369 (100%) Median (IQR)	Nutritional Status Based on Body Mass Index			
		Normal *n* = 184 (50.4%) Median (IQR)	Overweight *n* = 96 (26.3%) Median (IQR)	Obesity *n* = 85 (23.3%) Median (IQR)	*p*- Value ^†^
Energy, kcal	515 (366, 693)	524 (397, 693)	467 (340, 702)	489 (354, 664)	0.425
Contribution to energy/day (%) *	32.6 (23.2, 43.9)	33.2 (25.1, 43.9)	29.6 (21.5, 44.5)	31.0 (22.4, 42.0)	
Macronutrients distribution (%)					
Proteins	11.7 (9.5, 13.5)	11.8 (9.5, 13.3)	12.0 (9.8, 13.9)	11.4 (9.1, 13.5)	0.347
Carbohydrates	59.2 (52.8, 65.1)	58.5 (51.8, 65.2)	59.5 (54.8, 64.1)	60.5 (51.4, 65.9)	0.429
Fats	28.9 (24.1, 33.7)	29.2 (25.7, 34.5)	28.8 (23.3, 33.2)	28.4 (20.8, 34.4)	0.278
Cost/100 kcal (Mexican pesos)	4.0 (3.4, 4.6)	4.0 (3.4, 4.6)	4.2 (3.6, 4.8)	3.8 (3.3, 4.5)	0.046 ^‡^
Boys (*n*, %)	164 (44.4)	69 (37.5)	45 (46.9)	48 (56.5)	
Energy, kcal	535 ^§^ (376, 745)	557 ^§^ (439, 744)	517 (330, 769)	510 (347, 709)	0.203
Contribution to energy/day (%) *	33.9 (23.8, 47.2)	35.3 (27.8, 47.1)	32.7 (20.9, 48.7)	32.3 (21.9, 44.9)	
Girls (*n*, %)	205 (55.5)	115 (62.5)	51 (53.1)	37 (43.5)	
Energy, kcal	476 (364, 669)	487 (374, 673)	451 (347, 697)	467 (372, 638)	0.887
Contribution to energy/day (%) *	30.1 (23.1, 42.4)	30.8 (23.7, 42.6)	28.5 (22.0, 44.2)	29.6 (23.6, 40.4)	

* Percentage of energy contribution of kcal ingested during school hours compared to daily energy intake recommended for Mexican schoolchildren. ^†^ Kruskal–Wallis test for medians of kcal, for medians of contribution to energy/day, and for medians of macronutrient distribution by nutritional diagnostics. ^‡^ Dunn’s test of multiple comparisons, overweight vs. normal and overweight vs. obesity: *p* < 0.05. ^§^ Wilcoxon rank sum test for kcal by sex, all children: *p* = 0.051, normal children: *p* = 0.018. IQR: interquartile range.

**Table 3:** Calorie consumption and cost/100 kcal by nutritional status and food origin in schoolchildren in Mexico City.

Food Origin	All Children n = 369 (100%) Median (IQR)	Nutritional Status Based on Body Mass Index			
		Normal n = 184 (50.4%) Median (IQR)	Overweight n = 96 (26.3%) Median (IQR)	Obesity n = 85 (23.3%) Median (IQR)	*p*- Value ^†^
1. Home or school (n, %)	35 (9.5)	16 (8.7)	11 (11.5)	7 (8.2)	
Energy (kcal)	329 ^‡^ (223, 476)	370 ^‡^ (225, 531)	328 ^‡^ (203, 554)	325 ^‡^ (299, 391)	0.805
Contribution to energy/day (%) *	20.8 (14.1, 30.1)	23.4 (14.2, 33.6)	20.8 (12.9, 33.8)	20.6 (19.0, 24.7)	
Cost/100 kcal (Mexican pesos)	3.8 (3.1, 4.5)	3.9 (3.6, 5.6)	4.3 (3.1, 4.5)	2.9 (2.3, 4.1)	0.053
2. School and outside school (n, %)	42 (11.4)	25 (15.2)	8 (8.3)	6 (7.0)	
Energy (kcal)	465 ^‡^ (364, 553)	468 ^‡^ (353, 537)	428 ^‡^ (361, 518)	564 (372, 722)	0.483
Contribution to energy/day (%) *	29.4 (23.1, 35.0)	29.6 (22.4, 34.0)	27.1 (22.9, 32.8)	35.7 (23.6, 45.7)	
Cost/100 kcal (Mexican pesos)	4.1 (3.2, 4.6)	4.0 (3.0, 4.4)	4.2 (3.7, 5.1)	4.3(3.9, 4.8)	0.314
3. Home and school or home and outside of school (n, %)	97 (26.3)	45 (24.5)	25 (26.0)	27 (31.8)	
Energy (kcal)	451 ^‡^ (319, 606)	484 ^‡^ (355, 629)	387 ^‡^ (303, 564)	424 ^‡^ (286, 517)	0.218
Contribution to energy/day (%) *	28.5 (20.2-38.4)	30.7 (22.5, 39.8)	24.5 (19.2, 35.7)	26.8 (18.1, 32.8)	
Cost/100 kcal (Mexican pesos)	3.7 (3.3, 4.6)	3.7 (3.1, 4.3)	4.0 (3.7, 5.1)	3.6(3.2, 4.8)	0.186
4. Home, school, and outside school (n, %)	195 (52.9)	95 (51.6)	52 (54.2)	45 (52.9)	
Energy (kcal)	597 (428, 781)	610 (449, 800)	585 (397, 840)	587 (430, 737)	0.918
Contribution to energy/day (%) *	37.8 (27.1, 49.5)	38.7 (28.4, 50.7)	37.0 (25.1, 53.2)	37.2 (27.2, 46.6)	
Cost/100 kcal (Mexican pesos)	4.1 (3.6, 4.7)	4.0 (3.5, 4.7)	4.0 (3.5, 5.1)	3.8(3.5, 4.6)	0.242
*p*-Value of kcal by food origin ^†^	<0.001	<0.001	<0.001	<0.001	
*p*-Value food cost by food origin ^†^	0.139	0.113	0.781	0.085	

* Percentage of kcal ingested during school hours in relation to daily kcal recommended for Mexican schoolchildren. ^†^ Kruskal–Wallis test for kcal, for contribution to energy/day recommended, and for cost/100 kcal. ^‡^ Dunn’s test of multiple comparisons for kcal by origin in all children: one vs. two, one vs. three, one vs. four, two vs. four, three vs. four, *p* < 0.05; in normal children: one vs. three, one vs. four, two vs. four, three vs. four, *p* < 0.05; in overweight children: one vs. four, two vs. four, three vs. four, *p* < 0.05; and in obese children: one vs. two, one vs. four, three vs. four, *p* < 0.05. Comparison between food origin and nutritional status based on BMI categories; chi^2^ = 6.42, *p* = 0.378. IQR: interquartile range.

**Table 4:** Groups of foods * with the greatest consumption during school hours in a week, according to the food origin, in schoolchildren in Mexico City.

Total Foods			Foods from Home			Foods from School			Food from Outside School		
	Children *n* = 369	%		Children *n* = 307	%		Children *n* = 351	%		Children *n* = 251	%
Fruits	290	78.6	Fruits	190	61.9	Popcorn	179	51.0	Chips	84	33.5
Chips/popcorn	224	60.7	Sandwich	103	33.5	Fruits	172	49.0	Fruits	80	31.9
Sorbet	178	48.2	Torta ^b^	93	30.2	Tacos ^a^	160	45.6	Sugared drink	48	19.1
Tacos ^a^	170	46.1	Sugared drink	76	24.8	Sorbet	130	37.0	Vegetables	41	16.3
Vegetables	168	45.5	Fermented milk drink	66	21.5	Sweets/candy	118	33.6	Sweets/candy	37	14.7
Sugared drink	161	43.6	Bread	45	14.7	Vegetables	112	31.9	Ice cream	34	13.5
Sweets/candy	160	43.4	Cookies	40	13.0	Dessert	101	28.8	Cookies	33	13.1
Dessert	153	41.5	Yogurt	35	11.4	Ice cream	99	28.2	Sorbet	25	10.0
Cookies	129	35.0	Vegetables	34	11.1	Seeds	78	22.2	Fermented milk drink	25	10.0
Torta ^b^	118	32.0	Gelatin	30	9.8	Cookies	57	16.2	Bread	24	9.6

* The foods reported by the children were grouped by similar nutritional characteristics, and the most frequently reported 10 groups of foods by the origin are described. ^a^ Tacos: rolled corn tortilla with some food inside. ^b^ Torta: salty bread that is filled with various foods.

**Table 5:** Nutritional characteristics and cost of the 10 most consumed food groups during school hours, by food origin, in schoolchildren in Mexico City.

Food Groups	Portion g		Kilocalories per Portion		Cost (Pesos)per Portion		Cost (Pesos) per g	Caloric Density kcal/g	Proteins	Carbohydrates	Fats	Fiber
	Median	IQR	Median	IQR	Median	IQR	Median	Median	g per Portion (Median)			
Foods brought from home												
Fruits	104	64, 108	55	30, 62	2.6	1.9, 4.2	0.04	0.5	0.4	14.5	0.2	1.6
Sandwich	113	101, 130	303	292, 340	7.3	5.4, 8.9	0.06	2.9	11.4	24.4	16.2	1.0
Torta ^a^	129	114, 155	328	307, 395	6.9	4.9, 8.7	0.05	2.6	12.9	32.7	15.2	1.1
Sugared drink	300	250, 500	88	66, 116	5.0	4.5, 7.3	0.01	0.2	0.0	16.5	0.0	0.0
Fermented milk drink	80	80, 80	55	55, 55	5.4	5.4, 5.4	0.07	0.7	1.0	12.7	0.0	0.0
Bread	60	50, 60	224	185, 244	6.0	4.9, 6.0	0.10	3.7	3.6	28.4	10.9	0.6
Cookies	37	24, 60	150	105, 284	3.8	1.9, 6.0	0.07	4.6	2.0	25.9	6.0	0.6
Yogurt	240	240, 240	175	175, 175	7.0	7.0, 7.0	0.03	0.7	7.5	35.0	2.5	0.0
Vegetables	80	55, 140	22	11, 53	1.8	0.9, 1.8	0.02	0.4	1.0	4.9	0.1	2.1
Gelatin	135	135, 135	84	84, 84	3.2	3.2, 3.2	0.02	0.6	1.7	19.2	0.0	0.0
Foods bought at school												
Popcorn	37	22, 46	132	78, 198	5.0	5.0, 5.0	0.14	3.6	3.1	21.1	5.9	2.3
Fruits	89	77, 180	48	29, 79	5.0	4.2, 5.0	0.03	0.5	0.6	12.4	0.1	1.2
Tacos ^b^	89	72, 153	186	126, 297	5.0	5.0, 10.0	0.07	1.9	7.3	22.8	4.8	1.5
Sorbet	46	46, 46	40	40, 40	5.0	5.0, 5.0	0.11	0.9	0.0	10.0	0.0	0.0
Sweets/candy	8	7, 14	39	26, 43	3.0	3.0, 5.0	0.38	3.9	0.7	6.5	0.0	0.1
Vegetables	154	105, 155	21	14, 59	5.0	5.0, 5.0	0.03	0.4	1.1	4.8	0.0	2.2
Dessert	50	40, 106	118	102, 153	5.0	5.0, 5.0	0.10	2.5	2.6	20.8	2.2	0.5
Ice cream	105	73, 105	170	168, 170	5.0	5.0, 5.0	0.05	1.6	5.0	27.0	4.5	0.0
Seeds	41	36, 53	41	37, 54	5.0	5.0, 5.0	0.11	1.0	12.6	28.3	15.7	3.1
Cookies	32	14, 40	146	63, 186	5.0	2.5, 5.0	0.13	4.6	1.7	21.5	4.0	0.8
Foods bought outside of school												
Chips	35	35, 35	200	193, 200	8.4	6.7, 8.6	0.23	5.5	2.5	18.8	12.5	1.3
Fruits	84	64, 134	46	30, 60	2.6	1.8, 4.3	0.04	0.5	0.6	12.0	0.1	1.3
Sugared drink	300	250, 500	88	66, 160	5.0	4.5, 8.0	0.01	0.4	0.0	22.0	0.0	0.0
Vegetables	105	60, 140	27	11, 53	1.8	1.3, 1.8	0.01	0.4	1.0	6.2	0.1	3.4
Sweets/candy	12	6.5, 20	43	26, 80	3.1	2.0, 6.0	0.45	3.9	0.7	7.4	0.0	0.1
Ice cream	89	73, 105	168	110, 170	10.0	5.0, 10.0	0.09	1.6	4.0	26.5	4.5	0.0
Cookies	30	18, 60	138	75, 260	4.9	1.2, 9.0	0.14	4.6	2.0	22.0	6.0	1.0
Sorbet	46	46, 190	40	40, 243	10.0	10.0, 10.0	0.22	0.9	0.0	10.0	0.0	0.0
Fermented milk drink	80	80, 80	55	55, 55	5.4	5.4, 5.4	0.07	0.7	1.0	12.7	0.0	0.0
Bread	60	60, 60	240	224, 260	6.0	6.0, 6.0	0.10	4.1	2.8	28.4	12.6	0.6

^a^ Torta: salty bread that is filled with various foods.^b^ Tacos: rolled corn tortilla with some food inside.

## Data Availability

The data presented in this study are available on request from the corresponding author. The data are not publicly available.
